# Extending dental nurses’ duties: a national survey investigating skill-mix in Scotland’s child oral health improvement programme (Childsmile)

**DOI:** 10.1186/1472-6831-14-137

**Published:** 2014-11-25

**Authors:** Wendy Gnich, Leigh Deas, Sarah Mackenzie, Jacqueline Burns, David I Conway

**Affiliations:** Community Oral Health Section, Glasgow Dental School, Faculty of Medicine, University of Glasgow, 378 Sauchiehall Street, Glasgow, G2 3JZ UK; Public Dental Services, NHS Lanarkshire, Buchanan Centre, 1st Floor, Main Street, Coatbridge, ML5 3BJ UK; Salaried and Community Primary Care Dental Services, NHS Ayrshire and Arran, North West Kilmarnock Area Centre, Western Road, Kilmarnock, KA3 1NQ UK

**Keywords:** Childsmile, Children, Dental practice, Dental nurses, Dental caries, Oral health, Prevention, Skill-mix, Role-supplementation

## Abstract

**Background:**

Childsmile is Scotland’s national child oral health improvement programme. To support the delivery of prevention in general dental practice in keeping with clinical guidelines, Childsmile sought accreditation for extended duty training for dental nurses to deliver clinical preventive care. This approach has allowed extended duty dental nurses (EDDNs) to take on roles traditionally undertaken by general dental practitioners (GDPs). While skill-mix approaches have been found to work well in general medicine, they have not been formally evaluated in dentistry. Understanding the factors which influence nurses’ ability to fully deliver their extended roles is necessary to ensure nurses’ potential is reached and that children receive preventive care in line with clinical guidance in a cost-effective way. This paper investigates the supplementation of GDPs’ roles by EDDNs, in general dental practice across Scotland.

**Methods:**

A cross-sectional postal survey aiming to reach all EDDNs practising in general dental practice in Scotland was undertaken. The survey measured nurses’: role satisfaction, perceived utility of training, frequency, and potential behavioural mediators of, preventive delivery. Frequencies, correlations and multi-variable linear regression were used to analyse the data.

**Results:**

Seventy-three percent of practices responded with 174 eligible nurses returning questionnaires. Respondents reported a very high level of role satisfaction and the majority found their training helpful in preparing them for their extended role. While a high level of preventive delivery was reported, fluoride vanish (FV) was delivered less frequently than dietary advice (DA), or oral hygiene advice (OHA). Delivering FV more frequently was associated with higher role satisfaction (p < 0.001). Those nurses who had been practising longer reported delivering FV less frequently than those more recently qualified (p < 0.001). Perceived difficulty of delivering preventive care (skills) and motivation to do so were most strongly associated with frequency of delivery (p < 0.001 for delivery of FV, DA and OHA).

**Conclusions:**

This study has provided insight into EDDNs’ experiences and demonstrates that with appropriate training and support, EDDNs can supplement GDPs’ roles in general dental practice in Scotland. However, some barriers to delivery were identified with delivery of FV showing scope for improvement.

**Electronic supplementary material:**

The online version of this article (doi:10.1186/1472-6831-14-137) contains supplementary material, which is available to authorized users.

## Background

Despite recent changes for the better, improving the oral health of children living in Scotland is a challenge with wide inequalities persisting. Although approximately 67% of Primary 1 (5 year old) children in Scotland have no obvious tooth decay, there is a strong socio-economic gradient with 49.5% of those living in the most deprived geographical areas having observable caries by the time they go to school [1]. Moreover, dental registration rates are relatively low for very young children with only 47.2% of 0–2 year olds currently registered [2] and limited preventive dental care being delivered to children registered with a National Health Service (NHS) dentist [3]. To address these issues, in 2005, a national oral health programme for children living in Scotland (Childsmile) was funded by the Scottish Executive [4]. Childsmile aims to improve the oral health of Scotland’s children through a comprehensive, longitudinal, pathway of care delivered in clinical and community settings [5].

‘Childsmile Practice’, a key component of the Childsmile programme, promotes a shift from ‘reactive management’ to ‘anticipatory care’ [5]. Parents are encouraged to register their child with a dentist from six months of age. Once registered with a general dental practice, Childsmile advocates that families receive tailored preventive care comprising tailored dietary advice (DA), oral hygiene advice (OHA), and the clinical intervention of fluoride varnish application (FVA). Childsmile’s recommendations are in keeping with recent clinical guidelines stating that all children (irrespective of caries risk status) should receive, OHA at least once per year (with hands-on toothbrushing instruction in the early stages of providing care), DA at least once per year and if aged over two years (and not contra-indicated) fluoride varnish (FV) applied to their teeth at least twice a year in practice [6, 7].

In 2005, consultation during early programme development between Childsmile’s executive planning committee, public and oral health specialists (including NHS consultants in dental public health) and the General Dental Council, raised the concern that general dental practitioners’ (GDPs) capacity could potentially act as a barrier to the delivery of preventive care as recommended by the programme. This recognition led to Childsmile being instrumental in extending dental nurses’ roles and promoting a skill-mix approach within general dental practice [8].

Skill-mix can be defined as the mix of skills and staff required to deliver a service. This can be achieved by enhancing the role or skills of a particular staff group, substituting one staff member for another, delegating tasks previously performed by one staff group to another or introducing new types of staff [9]. Skill mix can be categorised into ‘role supplementation’ where less qualified staff members provide services that complement the activity of more qualified health-care workers and ‘role substitution’ where they replace the role of their senior colleagues [10, 11]. Brocklehurst and Tickle [12] explain that the latter can only occur and be cost-effective, if the more qualified colleague ceases to undertake the delegated tasks [12]. As Childsmile utilises the whole dental team, extended duty dental nurses (EDDNs) are trained to supplement the activity of more highly skilled (and costly) GDPs.

The use of skill-mix in the UK was first implemented within general medicine in the 1970’s [13, 14], although its uptake in dentistry has been relatively slow [15–17]. Factors affecting the broader use of the whole dental team include: the extent of clinical activity that is permitted by the dental regulator, financial incentives within the payment system, availability of appropriate training for dental care professionals (DCPs), lack of clarity over the roles that DCPs can perform and the perceived threat to the dentists’ autonomy [12, 15, 16, 18].

While EDDNs now operate throughout the NHS in the UK, when it was implemented in 2006, Childsmile was ground-breaking in its development of accredited training for EDDNs, enabling FVA under clinical supervision for the first time, an activity traditionally undertaken by the GDP. This training built on the changes that had been made by the dental regulator in 2002, which extended the range of duties which could be undertaken by DCPs [17, 19]. Childsmile’s extended training for dental nurses was developed in partnership with and is delivered by NHS Education Scotland.

The literature suggests that skill-mix can have the potential to improve patient access and patient and professional satisfaction [20–22]. However, for such benefits to be realised, barriers to implementation must be overcome: ensuring an education and training programme that is ‘fit for purpose’ [14, 21], increasing the supervision of staff [20, 21] and ensuring good human resource management to avoid unintended consequences such as increased occupational stress and lowered morale [9, 14, 23]. These findings, coupled with the scale and innovative nature of the roll out of a new approach that embraces skill-mix within general dental practice across Scotland, pointed to the need for formal evaluation of the EDDN role.

This paper explores the extent to which EDDNs are contributing to skill-mix within dental practice across Scotland through supplementation of GDPs’ roles. Key research questions are whether EDDNs are satisfied with their role, whether they feel adequately equipped by the training they receive and to what extent they are delivering preventive care (DA, OHA and FVA) to their child patients. Potential barriers and facilitators to EDDNs’ delivery of preventive care to children in Scottish dental practices are investigated. Learning about nurses’ experiences of applying new skills within practice, and the factors which facilitate or hinder their use, is a necessary step to ensuring that nurses’ potential is reached and that children receive preventive care in line with clinical guidance in a cost-effective way.

## Methods

A cross-sectional postal survey aiming to reach all accredited EDDNs currently working in general dental practice in Scotland was undertaken.

### Consent and ethical review

Respondents were informed as to how the information they provided would be used and consented through participation. The West of Scotland Research Ethics Service advised that under the terms of the governance arrangements for research ethics committees in the United Kingdom, NHS ethical review was not required. Glasgow University Medical Faculty Ethics committee approved the evaluation of Childsmile of which this study comprises one component. NHS clinical governance approval was obtained.

### Sample

At the time of study, there was no record of accredited, or currently practising, EDDNs which could be used as a sampling frame. 387 dental practices had agreed to be part of Childsmile (as at 31st December 2010), affording a sampling frame at the level of practice. All Childsmile practices were asked to participate.

### Measures

A bespoke questionnaire was developed to measure EDDNs’ satisfaction with their role, their perceived utility of the training they received, their frequency of delivering preventive care (DA, OHA and FVA) and factors hypothesised *a priori* to potentially mediate the extent to which nurses were employing their new skills in practice. Canvassing of expert opinion (including behavioural experts, dental clinicians, dental public health specialists, health services researchers and Childsmile proponents) and a review of relevant literature suggested the factors hypothesised *a priori* that were potentially related to EDDNs’ delivery of preventive care. The questionnaire was piloted (face-to-face) on a convenience sample of dental nurses who had just completed Childsmile’s extended duty training. Minor modifications at this stage ensured clarity of wording, and appropriate structure and length. A copy of the questionnaire is available from the corresponding author.

#### Demographic characteristics

*Age of dental nurse:* an interval measure (in years).

*Time qualified as a dental nurse:* an interval measure (in years).

*Time practising as a Childsmile EDDN:* an interval measure (in years).

*Practice setting*: a categorical measure indicating where the respondent worked (General Dental Service/Community Dental Service/Salaried Dental Service or a combination of settings).

*Number of dentists in practice*: an interval measure indicating number of professionals in a practice.

Five additional measures were obtained from the Management Information Dental Accounting System Database (MIDAS), NHS Scotland Information Services Division (ISD) for all sampled practices: General Dental Service status (General Dental Service or Community Dental Service/Salaried Dental Service), Childsmile delivery status (whether the practice had claimed for Childsmile treatment in the last six months), national Scottish Index of Multiple Deprivation [24] of practice (quintile 1–5), urban/rural classification of practice location (urban/small town/rural) and the number of dentists working at the practice. Two measures (Childsmile delivery status and number of working dentists) were only available for General Dental Service practices. Data was extracted for the six month period closest to survey administration (1st August 2010 to 31st December 2010).

#### Job satisfaction

*Satisfaction with job in general/Satisfaction with role as an EDDN:* Items were measured using a Likert type scale (not at all satisfied, not very satisfied, somewhat satisfied, very satisfied, completely satisfied).

#### Utility of training

The utility of the basic Childsmile training course in preparing dental nurses for their role as an EDDN was measured on a Likert type scale (not at all helpful, not very helpful, somewhat helpful, very helpful, extremely helpful).

#### Preventive delivery

*Self-reported frequency of DA, OHA and FVA:* The frequency of EDDNs delivering DA, OHA and FVAs to child patients was measured using a Likert type scale (never, rarely, sometimes, often, always). For DA and OHA, frequency of delivery was asked about for two clinically relevant age-based subgroups (children under two years of age and those aged two and above). Frequency of FVA was only questioned for children two years or older since varnish application is not recommended for younger age groups.

#### Potential behavioural mediators of preventive delivery

Six variables were assessed: Knowledge; skills; social support; motivation; resources; and confidence. In order to adequately represent the conceptual complexity of knowledge, social support, motivation and resources, within a single variable, nurses’ responses to multiple items were combined into ‘scale scores’. The items comprising each variable are presented in Additional file 1.

#### Open questions

Respondents were asked the following free text questions: whether there were any topics that would have been useful, or should have been expanded upon, within their training and to list the three things that made it easier, and made it harder for them, to carry out their role as an EDDN. They were also prompted to provide any further comments on their experience as an EDDN which they thought may be useful to the aims of the research.

### Procedure

Dental Practices were sent a postal questionnaire (February 2011) for completion by all trained EDDNs currently delivering Childsmile at that location. Since it was possible that some sampled practices did not employ a trained dental nurse (as Childsmile was being delivered by a dentist or other Allied Health Professionals), or alternatively that the practice had not yet started delivering the programme, a return slip to indicate ineligibility was provided alongside the questionnaire. Instruction was given that all accredited EDDNs at a practice should complete the questionnaire (and contact details given to request additional questionnaires). Trained nurses who had not yet started to deliver Childsmile sessions were requested only to provide demographic information. Two follow-up mailings were undertaken in March and May 2011.

### Analysis

Data was entered into and statistical analyses carried out in SPSS (V21). A 10% random sample of questionnaires was entered into SPSS by two operators, and the datasets compared in order to identify any systematic data entry errors. Valid range and logic checks were conducted on the full data set. Frequencies were produced for all variables and means and standard deviations for interval variables. Chi-square and independent t-tests were used to examine differences between responding and non-responding practices. Where responses to more than one question were combined to create a single variable (representing a potential behavioural mediator of preventive delivery), individual responses on each item were scored positively, summed and an average ‘scale score’ calculated for each respondent. Item analysis was undertaken to consider if individual items should be included in the scale. Cronbach’s alpha coefficient (an estimate of internal consistency) was calculated to test for reliability of the items within each scale [25]. Correlation co-efficients (Pearson’s r or Spearman’s rho) appropriate to each item’s level of measurement were used to examine the uni-variate associations between demographic characteristics, job satisfaction, utility of training and potential behavioural mediators of preventive delivery with the five measures of frequency of preventive delivery. In order to identify those potential behavioural mediators of preventive delivery (theoretically amenable to change), that were most strongly and independently associated with the preventive outcomes, multivariable linear regression models were run using a stepwise variable selection method with a strict p < 0.001 for model entry. A categorical variable representing respondents’ practice was offered to the model as an explanatory (or independent) variable alongside potential behavioural mediators in order to control for the possibility that respondents from the same practice could have more similar patterns of delivery than those from different practices. Open questions frequently produce multiple responses that require the creation of several variables to capture responses [23]. In this survey, free text responses to open questions were listed, then, to aid interpretation, those with common meaning were grouped into higher order thematic categories. For example, responses relating to the availability of staff, time, money, physical space and health promotion materials were coded to the theme ‘resources’. The number of responses coded to each ‘theme’ was then counted to give an indication of response frequency.

## Results

### The study population

#### Response

The response rate was calculated at the level of practice. Of 376 practices initially mailed, 196 (52.1%) returned at least one questionnaire: 167 (85.2%) responding practices returned one questionnaire, 26 (13.3%) practices two, two practices (1.0%) three questionnaires and one practice (0.5%) five questionnaires. A further 78 (20.7%) returned a slip indicating that they did not have a trained EDDN. Of those practices returning slips 40 (51%) were delivering Childsmile. Three (0.8%) practices were no longer at the sampled address, giving a response rate of 73.4%. 230 trained nurses returned questionnaires. Of these, 56 (24.3%) were not practising and provided only demographic information. Thus, 174 eligible nurses returned questionnaires to indicate that they were accredited and currently delivering Childsmile sessions.

#### Demographic characteristics

All respondents were female and in the age range 19–55 years. The majority qualified as a dental nurse at least eight years ago, although most had been trained and practising as an EDDN for only a year or two. The number of dentists, in the practices in which respondents were employed, ranged from one to thirteen. The majority of respondents worked in the General Dental Service. Nurse and practice characteristics are reported in Table 1.

**Table 1:** **Sample demographics (for responding EDDNs and the practices in which they are employed)**

	N = 174
	n (%)
**Age**	
19-29	71 (40.8)
30-49	89 (51.1)
50+	12 (6.9)
Missing	2 (1.1)
**Length of time practising (years)**	
Median	1.08 (0.5, 2.7)
Range	0 to 7.42
Mean (Standard Deviation)	1.74 (1.61)
Missing	19
**Length of time qualified (years)**	
Median	6.00 (2.83, 11.65)
Range	0 to 31.75
Mean (Standard Deviation)	8.62 (7.42)
Missing	22
**Practice setting**	
General dental service	151 (86.8)
Community dental service	8 (4.6)
Salaried dental service	12 (6.9)
Other	2 (1.1)
Community dental service & salaried dental Service	1 (0.6)
**Number of dentists in practice**	
1	18 (10.3)
2	36 (20.7)
3-5	89 (51.1)
6-13	25 (14.4)
Missing	6 (3.4)

#### Responding versus non-responding practices

No significant differences were found between responding and non-responding practices in relation to their: General Dental Service status (72.4% of General Dental Service practices responded compared to 78.7% of Community Dental Service or salaried practices: X^2^ = 1.02, df = 1, p = .312); Childsmile delivery status (67% of General Dental Service practices who had delivered Childsmile sessions in the six months prior to delivery responded compared to 75% of those that had not); National Scottish Index of Multiple Deprivation (72.0%, 76.1%, 76.8% of practices with a Scottish Index of Multiple Deprivation quintile 1–2, Scottish Index of Multiple Deprivation quintile 3 and Scottish Index of Multiple Deprivation quintile 4–5 postcode respectively: X^2^ = 2.32, df = 4, p = .677); Urban/rural classification (71.3%, 78.1% and 87% of practices respectively in urban, small town and rural locations responded: X^2^ = 3.53, df = 2, p = 0.17) or the number of practising dentists [Mean = 4.10 (Standard Deviation = 2.40) for responders compared to Mean = 3.85 (Standard Deviation = 2.26) for non-responders: t = -0.845, df = 310, p = .399].

#### Role satisfaction

**Figure 1:**
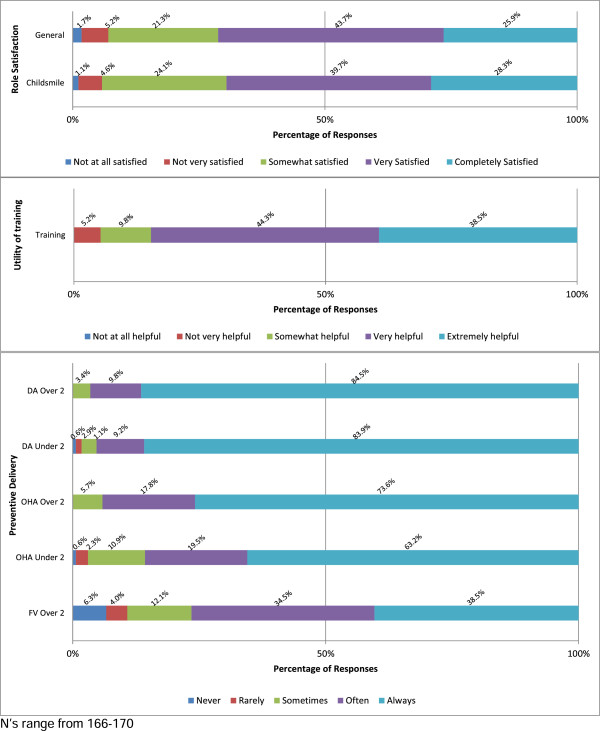
**Distribution of EDDNs’ role satisfaction, perceived utility of training and preventive delivery.**

Over two-thirds of respondents were completely (45, 25.9%) or very (76, 43.7%) satisfied with their job in general. Similar proportions were completely (49, 28.2%) or very (69, 39.7%) satisfied with their role as an EDDN. See Figure 1.

Free text responses suggested that delivering extended duties contributed to nurses’ job satisfaction:

“[I’ve] really enjoyed being a Childsmile nurse (EDDN) enjoy it more than just being a dental nurse.” DN43

Similarly, another respondent stated:

“I enjoy my work as a Childsmile nurse (EDDN), I feel more fulfilled within my job” DN21.

However, several respondents noted that their increased responsibilities were not reflected in an increase in pay:

“I do think we should be paid more to reflect the work we now do as Childsmile nurses (EDDNs).” DN3

#### Utility of training

Overall, respondents thought the Childsmile training they undertook was extremely (67, 38.5%) or very (77, 44.4%) helpful in preparing them for their role as an EDDN (See Figure 1). Several respondents commented on the benefits of the training they received:

“I have learned so much after the training and I am better prepared to answer questions that people might ask. I knew absolutely nothing about breastfeeding and weaning and really enjoyed finding out about this.” DN44

When asked what additions to training would have been beneficial, the most common response was ‘more weaning and dietary advice’. Several respondents commented that the training included a lot of theory and that they would have benefited from more hands-on practical experience:

“When attending the Childsmile course there was a lot of theory… It would have been helpful to have more practice as well as theory.” DN28

This was particularly in relation to FVA and toothbrushing demonstration:

“The application of FV on monkey heads (models) wasn’t very helpful. It would have been better to have been able to go out with a Childsmile nurse (EDDN) to the nurseries and schools to get a better insight on how the children behave when having FVA.” DN48

The quote above also highlights another common theme, the need for training to focus more on how nurses can successfully engage and communicate with children and parents, particularly ‘awkward parents’. Others mentioned how additional time spent on ‘role-plays’ or ‘mock sessions’ could have furthered this aim.

#### Preventive delivery

Figure 1 presents the self-reported frequency of delivery (stratified by patient age) for each preventive intervention. On the whole a very high level of preventive delivery was reported.

For children two years and above, DA (94.3% of nurses reported delivering ‘always’ or ‘often’) and OHA (91.4% of nurses reported delivering ‘always’ or ‘often’) were delivered more frequently than FV (73% reported delivering ‘always’ or ‘often’). The frequency of delivering DA to younger children (93.1% reported delivering ‘always’ or ‘often’) was similar to the frequency of information given to those over two years. OHA was delivered to younger children (82.7% reported delivering ‘always’ or ‘often’) slightly less often than their older counterparts. While FVA lagged behind the delivery of both DA and OHA, it is notable that almost three quarters of respondents reported delivering FV ‘always’ or ‘often’.

#### Demographic characteristics associated with preventive delivery

Demographic characteristics were not associated with the frequency of delivering DA or OHA for children aged below two or two years and above. However, dental nurses’ age (r = -0.197, p = <0.05), time practising as an EDDN (r = -0.370, p = <0.001) and the number of dentists in the practice (r = -0.219, p = <0.01) were associated with frequency of FVA. Older and longer practising nurses applied varnish less frequently as did those working in a practice with more dentists. See Table 2.

**Table 2:** **Pearson’s correlation coefficients (r) for potential associates of preventive delivery by EDDNs**

	DA under 2	DA over 2	OHA under 2	OHA over 2	FVA over 2
*Demographics*					
Nurses’ age	-.040	-.051	-.037	-.060	-.197*
Years qualified as dental nurse	.054	-.025	.013	.000	-.117
Years practising as dental nurse	.132	.091	.092	.034	-.370***
Number of dentists in practice	-.046	.107	-.045	-.003	.219**
Practice setting (GDS & Other)	.012^Ϯ^	.054^Ϯ^	-.041^Ϯ^	.006^Ϯ^	-.089^Ϯ^
*Role Satisfaction*					
Satisfaction with job in general	.082	.036	.054	.044	.092
Satisfaction with EDDN role	.052	.063	.088	.059	.278***
*Utility of Training*	.019	.113	.060	.040	.300***
*Other Behavioural Mediators*					
Knowledge	.141	.174*	.071	.129	.284***
Skills:					
Skills (DA)^§^	.287***	.287***			
Skills (OHA)^§^			.298***	.298***	
Skills (FV)^§^					.504***
Social Support	.115	.123	.079	.129	.205**
Motivation:					
Motivation (DA)^§^	.315***	.415***			
Motivation (OHA)^§^			.302***	.356***	
Motivation (FV)^§^					.610***
Resources	.065	-.024	-.012	-.031	.086
Confidence	.193*	.226**	.019	.070	.269**

*< 0.05 (2-tailed) **< 0.01 (2-tailed) ***< 0.001 (2-tailed).

^Ϯ^Spearmans’ Rho.

^§^The item composition of these scales is tailored for the appropriate preventive outcomes (Dietary advice (DA), Oral hygiene advice (OHA) and fluoride varnish application (FVA).

N’s range from 151–174.

#### Satisfaction with role and preventive delivery

Role satisfaction (in general and as an EDDN) was not associated with the delivery of DA or OHA for either patient group. However, satisfaction as an EDDN was associated with the frequency of FVA (r = 0.278, p = <0.001). Dental nurses who delivered FV more frequently were more satisfied with their role as an EDDN. Role satisfaction is presented in Table 2.

#### Utility of training and preventive delivery

Similar to role satisfaction, perceived utility of training was associated with frequency of FVA but not DA or OHA. Those who found the training more helpful were more likely to apply varnish (r = 0.300, p = <0.001).

#### Potential behavioural mediators associated with preventive delivery

##### Analysis of quantitative variables

Table 3 shows the internal consistency and descriptive statistics for each potential behavioural mediator (knowledge, skills, social support, motivation, resources and confidence) associated with preventive delivery. Cronbach’s alpha ranged from 0.62 to 0.76, indicating high internal reliability of the measures.

**Table 3:** **Descriptive statistics for potential behavioural mediators of preventive delivery by EDDNs**

	Alpha	Range	Mean (SD)
Potential behavioural mediators			
Knowledge	0.713	2.67-5.00	4.68 (0.47)
Social support	0.648	1.33-5.00	3.70 (0.85)
Motivation			
Motivation (DA)	0.675	3.00-5.00	4.89 (0.34)
Motivation (OHA)	0.689	2.50-5.00	4.85 (0.35)
Motivation (FVA)	0.761	1.50-5.00	4.69 (0.63)
Resources	0.617	2.00-5.00	4.07 (0.72)
Skills			
Skills (DA)	na	2-5	4.73 (0.59)
Skills (OHA)	na	3-5	4.74 (0.51)
Skills (FVA)	na	1-5	3.93 (1.00)
Confidence	na	1-5	4.71 (0.60)

N’s range from 163–170.

The potential behavioural mediators associated with delivery varied in relation to which preventive outcome was measured (Table 2). ‘Skills’ and ‘motivation’ were both uni-variately associated with all preventive outcomes. The strength of association was stronger for FVA (r = 0.504, p = <0.001; r = 0.610, p = <0.001) than for DA (under two years: r = 0.287, p = <0.001; r = 0.315, p = <0.001; and, over two years: r = 0.287, p = <0.001; r = 0.415, p = <0.001) or OHA (under two years: r = 0.298, p=,0.001; r = 0.302, p = <0.001; and, over two years: r = 0.298, p = <0.001; r = 0.356, p = <0.001). Thus perceived difficulty of delivering the preventive activity, perceived importance of doing so and motivation were associated with frequency of preventive delivery. In contrast, respondents’ self-reported ‘confidence’ levels in relation to delivery were only associated with DA (under two years: r = 0.193, p = <0.05; over two years: r = 0.226, p = <0.01) and FVA (r = 0.269, p = <0.01). Respondents’ ‘knowledge’ of preventive delivery was also associated with DA, but only for patients over two years (r = 0.174, p = <0.05) and FVA (r = 0.284, p = <0.001). The strength of the associations with confidence and knowledge were weaker than for skills and motivation. Social support from colleagues was significantly associated with FVA (r = 0.205, p = <0.01) but not DA or OHA and resources (including time, space and equipment) were not significantly associated with frequency of any preventive measure.

Using a stepwise regression model, potential behavioural mediators amenable to change were entered into a multivariate analysis in order to explore interventions that may increase preventive delivery by EDDNs (See Table 4). Five models were run exploring the response (or dependent) variables DA (for children under two and two years and over), OHA (for children under two and two years and over) and FVA for children over two years of age. Knowledge, skills, social support, resources, motivation and confidence were offered to the model as potential explanatory (or independent) variables. A categorical variable representing respondents’ dental practice was also offered to the model, since respondents from the same practice may have had more similar patterns of delivery than those working in other practices. The variables that emerged as being independently associated with dental nurses’ delivery of preventive interventions were motivation and skills. Motivation entered four out of five models and was the variable which made the largest explanatory contribution to all the models it entered. Motivation did not independently predict the frequency of OHA given to those under two years of age. Skills entered three of the five models failing to add any additional explanation for frequency of DA for either patient group. All models were statistically significant, although the percentage of variance explained by variables entering the models ranged from just 9% for frequency of DA to those under two to over 48% for FVA.

**Table 4:** Results of explorative stepwise regression analyses including potential behavioural mediators associated with preventive delivery by EDDNs*

		Beta	95% CI for B		Adj R ^2^	df	f
			Lower	Upper			
DA Under 2	Motivation	.317	.285	.822	.094	1 (149)	16.61***
DA Over 2	Motivation	.407	.354	.756	.160	1 (150)	29.82***
OHA Under 2	Skills	.321	.274	.784	.097	1 (147)	16.84***
OHA Over 2	Motivation	.216	.085	.709			
					.106	2 (147)	9.81***
	Skills	.189	.022	.419			
FVA Over 2	Motivation	.526	.652	1.12			
					.484	2 (141)	67.98***
	Skills	.264	.133	.429			

***< 0.001.

N’s Range from 144–152.

*All behavioural mediators amenable to change (Knowledge, skills, social support, resources, motivation and confidence) were offered to the model in addition to a variable representing the dental practice in which responding EDDNs were employed.

##### Analysis of open responses

Thematic analysis of open questions asking nurses to list the three main barriers and facilitators to delivery of their extended duties provided further insight into potential mediators of preventive delivery. Free text responses suggested ‘resources’ were the most frequently listed facilitator (60.3%), support from colleagues the second most frequent (40.2%) and patient factors including ‘co-operative parents’ and ‘patients attending appointments’ the third (25.3%). An increased number of referrals/patients accounted for a further 10.9% of responses.

Turning to barriers, respondents listed ‘patient factors’ most frequently. This included ‘families failing to attend appointments’ (28% of responses); uncooperative/unmotivated parents (26%), nervous or aggressive parents (2.3%), uncooperative children (5.7%) and large families attending together (1.7%). Lack of patients/referrals was also listed by 6.3% of respondents. In keeping with the facilitators listed, ‘lack of resources’ (47%) and ‘lack of support from colleagues’ (10.3%) were also reported as barriers.

## Discussion

This study investigated the contribution of role supplementation by EDDNs, to skill mix in general dental practice across Scotland, by investigating: EDDNs’ satisfaction with their extended role, their views on the extended duty training they received and their experiences of delivering preventive care to children and their families.

On the whole, role supplementation was found to operate well, with EDDNs reporting frequent utilisation of their new skills in terms of delivering preventive care. This is in keeping with a growing body of literature suggesting that skill-mix, through extended roles, can make a positive contribution to the delivery of health-service provision [10, 11, 20–22, 26, 27]. Galloway et al. [22] found that dental care professionals (DCPs), including EDDNs, with appropriate training, can, extend into roles traditionally undertaken by the dentist, conduct oral health promotion and enhance productivity in a dental practice [26]. While this study of Childsmile’s EDDNs did not specifically measure the impact of their utilisation on productivity in general dental practice, a gap that has recently been acknowledged in the research literature [28], it concurs that with appropriate training EDDNs can deliver health promoting aspects of a dentist’s role in Scottish general dental practice.

EDDNs’ high role satisfaction, the positive association between frequency of delivering FV and role satisfaction and free text responses suggesting that attainment and delivery of new skills increased nurses’ job satisfaction are also in keeping with several studies reporting high employee satisfaction with extended roles [29–32].

Nonetheless, it is notable that FV was reported to be delivered less frequently than DA or OHA. This is perhaps unsurprising in light of recent research suggesting that FV is not being applied to children’s teeth in general dental practice in Scotland as often as current clinical guidelines would advocate [3, 33]. The Translational Research in a Dental Setting (TRiaDS) programme reported that a significant gap exists between optimal and actual practice for the prevention of child caries in Scotland with only 10% of GDPs surveyed reporting ‘always’ applying FV to their child patients’ teeth [3]. A much lower proportion, than the percentage of EDDNs who reported ‘always’ applying varnish in this Childsmile study. There are a several possible explanations for the high levels of preventive delivery reported in this study.

First, when interpreting the findings of the Childsmile study, it must be borne in mind that those practices sampled, had chosen to sign up to Childsmile. It is plausible that staff working in these ‘early adopting’ practices hold more favourable views of the operation of skill-mix in general dental practice than those employed in practices which did not ‘opt in’ to the programme. Now that Childsmile has been mainstreamed as the national NHS dental service for all children in Scotland, all practices are expected to deliver preventive care to child patients in line with Childsmile principles, not just those that sign up to the programme. A survey of EDDNs working in all GDP practices in Scotland may yield different results.

Second, within Scotland, at the time of this study, Childsmile practices were being paid a fee for preventive care, including FVA. Since October 2011 this financial incentive has been extended to all practices in Scotland through the Statement of Dental Remuneration. Payment may in part explain the high rates of preventive delivery reported in this study, although it does not explain why FVA is delivered less often than DA or OHA.

Third, in contrast to studies which have reported that lack of resources can act as a barrier to the use of skill mix [34, 35], the finding that resources, including time, staff capacity and equipment, were not associated with frequency of preventive delivery, may be a contributor to the high levels of preventive delivery reported in this study. The higher proportion of respondents who listed ‘resources’ as a factor which facilitated delivery of their EDDN role, than the proportion who listed ‘resources’ as a barrier, suggests that resources did not constrain delivery for the majority of EDDNs who responded. Carter et al. [36] also found that the majority of nurses attending a FV training scheme in England, had adequate access to resources and a large proportion reported no barriers to utilising their skills [36].

Despite high rates of preventive delivery, this study found that not all nurses are equally amenable to adopting and implementing new skills. Several factors were associated with the frequency of EDDNs’ preventive delivery. Nurses who had been practising longer, and to a lesser extent those who were older, were less likely to deliver FV as frequently as their colleagues. While research suggests that age is not a factor for resistance to change in skills or working practices, long-tenured staff may find it more difficult to adopt and implement new skills as they have invested more in their traditional role and working practices [37, 38].

EDDNs working in practices with a larger number of practising GDPs were also less likely to apply FV. This could perhaps reflect GDPs’ influence within the practice; in single-dentist or small practices it may be more likely that all dentists were signed up to Childsmile, with more variability in attitudes in a larger practice, where signing up to the programme was made by a lead dentist or a majority decision. Collegiate support for preventive delivery was associated with frequency of FVA and listed by respondents as both a barrier and enabler to their role. Since nurses are employed by GDPs, their employers’ attitudes towards role-supplementation will influence the extent to which dental nurses can implement their extended role. Interpretation of these findings requires further investigation.

EDDNs’ motivation to deliver preventive care (including the perceived importance of delivery) was consistently the most strongly associated potential mediator of frequency of delivery for all preventive behaviours investigated. Candell and Engstrom [39] found that motivating factors for dental hygienists included increased demand from patients and receiving recognition from patients of successful treatments, whilst barriers included patients cancelling appointments [39]. Similarly, this study found that cooperative parents and increased patient numbers were described as facilitators to the EDDN role and, uncooperative parents and families failing to attend appointments as barriers.

Finally, the perceived difficulty of delivering preventive care was also independently associated with frequency of delivery. The harder nurses perceived the task to be, the less likely they were to apply FV to their child patients’ teeth or to provide OHA, including demonstration of toothbrushing. That there was no significant association with frequency of delivering DA may reflect the differing task requirements; DA relies solely on verbal communication in contrast to the physical action required to demonstrate toothbrushing or apply FV. Additionally both toothbrushing demonstration and FVA require the nurse to physically interact with their child patient - a role they would not previously have undertaken.

Since role-supplementation requires the attainment of new knowledge and skills, it is unsurprising that our findings support Jacob et al.’s conclusion that as skill-mix increases there is a need to address how education affects competency and skill in the workforce [23]. While this study, like Carter et al. [36], found that training can prepare EDDNs for their new role, the most frequently suggested modifications to training are illuminating. Similarly to Hatim & Kendall, in their study of FV training for dental nurses [18], we found that suggestions point to more practical ‘hands-on’ experience as being beneficial. This was despite a formal requirement for Childsmile dental practices to provide EDDNs with an in-house mentor and for EDDNs to observe five FVAs administered by their mentor, complete ten FVAs and have their practice observed by their mentor prior to being certified as an EDDN [40].

That those nurses who found the training most useful were more likely to apply FV, and that nurses’ knowledge and confidence were related to frequency of FVA, further underscores the importance of fit for purpose training and skills attainment. Communications training aimed at increasing nurses’ confidence and skills in patient interaction may be helpful.

This study was a preliminary investigation of the contribution to skill-mix in general dental practice by a previously under-studied population (EDDNs). It provides insight into the working practices of a relatively new group of professionals and to the factors which influence key aspects of preventive oral health care delivery for children living in Scotland. To date, published papers focussing on the EDDN role have been entirely descriptive [18, 41]. However, Brocklehurst et al. have recently published a study protocol which aims to investigate the barriers and facilitators to role-substitution of DCPs in dental practices and to determine the most efficient model of role-substitution [28]. The authors point to the increased potential for use of skill mix in dental practice, in the future, explaining that projected improvement in the population’s oral health, suggests that if skill-mix does not become more widespread in dental practice that NHS resources will be unnecessarily “devoted to the use of highly skilled and paid workers to perform relatively simple tasks on an increasingly healthy practice population which less costly staff are competent to carry out” [28]. The most thorough published study of implementation of the EDDN role, undertaken by Carter et al. [36] formally investigated a FV training scheme for nurses within a single training centre in London, England. 36 nurses responded to an electronic survey which included questions about skills use following training. This study adds to current knowledge by uniquely exploring potential barriers and facilitators to preventive delivery by EDDNs both uni-variately and multi-variately and by focussing on the Scottish context. It also affords interpretation of findings based on a much larger sample size than previous studies of the EDDN population [18, 36, 41, 42] by sampling all practices in Scotland with the potential to employ a trained EDDN.

However, our inability to obtain an individual level sampling frame of all EDDNs delivering in dental practice in Scotland at the time of study and therefore to ascertain what percentage of those nurses responded may be considered a limitation. Although the practice-based response rate achieved is higher than the average for professional surveys of this type [32, 43, 44], those nurses who responded may have been more motivated and active than those who declined to respond. Nonetheless, responding and non-responding practices did not differ significantly in terms of demographic characteristics which may have suggested response bias.

Another potential limitation is the self-reported nature of the preventive delivery measures used in the study. No objective measure of EDDN’s delivery was available at the time of study.

Additionally, when interpreting the potential mediators of nurses’ delivery of preventive care it is important to bear the level of variance in terms of frequency of delivery in mind. For example, the stronger associations found between potential mediators and FVA as opposed to DA and OHA may be, at least partially, explained by the greater variance exhibited by this delivery outcome. Additionally, skills and motivation may have been more strongly associated with preventive outcomes than other potential behavioural mediators as they were measured specifically for each outcome, rather than in relation to delivery of the Childsmile role more generally, as was the case for the other behavioural mediators.

Nonetheless, this study has provided evidence as to the extent to which: the role supplementation approach, widely applied in the general medical context, has been successfully translated to the field of general dental practice (EDDNs are delivering important clinical prevention to Scottish children in line with national guidance), and provided insight into barriers and facilitators to this delivery. Further research could usefully seek deeper understanding of the factors influencing EDDNs’ ability to fully embrace an extended duty skill-mix role. A potentially productive direction may be to compare experiences of those EDDNs working in practices where preventive delivery is meeting national guidelines with those who are working in practices where guidance is less well implemented (although the extent to which practices implement these guidelines is not necessarily related to extent of delivery by EDDNs).

Qualitative methods could be employed to gain further understanding (from EDDNs and other members of the dental team) of the complex range of factors (and their interactions) which are likely to influence EDDNs’ ability to deliver preventive care, particularly FVA. Such a study may benefit from utilising an existing behavioural framework (e.g. Michie et al.’s Theoretical Domains Framework [45]) to ensure that all potential influences on behaviour are comprehensively covered and that findings can be translated into action to improve delivery of care in line with guidance. The theoretical domains framework consolidates existing psychological and organisational theory relevant to health practitioner clinical behaviour change [46, 47] and has been used previously within dentistry [48]. Since family factors were perceived to act as both barriers and enablers to extended duty delivery by dental nurses in this study, further research should also explore views on delivery of preventive care from the families’ perspective.

It is also acknowledged that investigating frequency of delivery, does not inform of the quality of that delivery, further evaluation is required to assess the quality of preventive delivery being delivered (by EDDNs and other dental team members) to children living in Scotland.

The lessons learned from this and proposed future studies would in addition to adding to the wider body of literature evaluating the translation of skill-mix policies (and their underlying theory into practice), provide a theoretical basis for intervening to improve operationalisation of the EDDN role, with a view to ultimately improving the oral health care that Scottish children receive from their dental team.

## Conclusions

The high levels of role satisfaction, perceived utility of training and extent of preventive care delivered by EDDNs found in this study demonstrate that with appropriate training and ongoing support from colleagues, EDDNs can supplement the role of GDPs in dental practice in Scotland and suggest a wider cultural shift towards preventive team-based approaches. Nurses embraced their new skills, and while lack of remuneration was noted by some, employing new skills contributed to job satisfaction. Nonetheless, some barriers to delivery were reported and delivery of FVA showed some scope for improvement. Future training for EDDNs could usefully include more opportunity to practise hands on delivery and focus on improving nurses’ communication skills and interaction with patients. Further, theoretically driven exploration of the barriers and enablers to nurses fulfilling their skills mix role (taking into account the views of professionals and the families they serve) and assessment of quality of delivery will afford the potential to undertake tailored interventions to support EDDNs in delivering preventive care in line with clinical guidelines in a cost-effective way.

## Authors’ information

WG is a research fellow in Dental Public Health and Evaluation at the Community Oral Health Section, University of Glasgow. LD and SM are Childsmile regional researchers employed by NHS Lanarkshire. JB is a dental foundation (2) trainee practitioner employed by NHS Ayrshire and Arran. DC is a clinical senior lecturer in Dental Public Health at the Community Oral Health Section, University of Glasgow. All authors, except JB are members of a larger evaluation team funded to undertake a comprehensive evaluation of Childsmile.

## Electronic supplementary material

Additional file 1: **Item composition of variables.** Description of data: Overview of individual items (as worded in survey questionnaire) comprising multiple-item scales created for use in the analysis. (DOCX 15 KB)

## Abbreviations

DA: Dietary advice
DCPs: Dental care professionals
EDDN(s): Extended duty dental nurse(s)
FV: Fluoride varnish
FVA(s): Fluoride varnish application(s)
GDPs: General dental practitioners
NHS: National Health Service
OHA: Oral hygiene advice.
