# Supplementary material for: Extending dental nurses’ duties: a national survey investigating skill-mix in Scotland’s child oral health improvement programme (Childsmile)
Source: BMC Oral Health. 2014 Nov 25;14:137. doi: 10.1186/1472-6831-14-137 (PMC4280710; doi:10.1186/1472-6831-14-137)
Supplement: Supplementary file 1 — Additional file 1: Item composition of variables. Description of data: Overview of individual items (as worded in survey questionnaire) comprising multiple-item scales created for use in the analysis. (DOCX 15 KB) [file 12903_2014_479_MOESM1_ESM.docx]

Additional File 1: Item composition of variables

| **Potential Associations** | **Sub-scale Items** |
| --- | --- |
| **Knowledge** | How Strongly Do you agree or disagree that you:  Understand the role of a Childsmile Nurse.  Understand the wider Childsmile programme.  Have the knowledge to deliver a Childsmile session.  (Rated Strongly Disagree to Strongly Agree) |
| **Skills:**  **Skills (DA)**  **Skills (OHA)**  **Skills (FV)** | To prevent caries in my Childsmile patients I think:  Providing dietary advice is not difficult to do.  Delivering toothbrushing demonstration is not difficult to do.  Applying fluoride varnish is not difficult to do.  (Rated Strongly Disagree to Strongly Agree) |
| **Confidence** | How Strongly Do you agree or disagree that you:  Were confident in delivering a Childsmile session.  (Rated Strongly Disagree to Strongly Agree) |
| **Social Support** | How strongly do you agree or disagree with the following statement:  Staff in my practice agree with all aspects of Childsmile Delivery.  How strongly do you agree or disagree that the following people support you as much as you need them to:  Childsmile Mentor.  Others in Practice.  Other dental nurses.  Dental Health Support Worker  Childsmile Coordinators  (Rated Strongly Disagree to Strongly Agree). |
| **Motivation:**  **Motivation (DA)**  **Motivation (OHA)**  **Motivation (FV)** | To prevent caries in my Childsmile patients I think:  Providing dietary advice is very important.  Providing dietary advice is something I am very motivated to do.  Delivering toothbrushing demonstration is very important.  Delivering toothbrushing demonstration is something I am very motivated to do.  Applying fluoride varnish is very important.  Applying fluoride varnish is something I am very motivated to do.  (Rated strongly disagree to strongly agree) |
| **Resources** | How strongly do you agree or disagree with the following statements?  I have enough time to see all my Childsmile patients as often as i would like.  I am not able to see Childsmile patients promptly when they require an appointment. **  I have enough time to cover everything I would like to within a Childsmile sessions,  I have appropriate space where I can deliver my Childsmile appointments.  I have adequate resources for my Childsmile sessions (i.e. toothbrush demonstration equipment)  (Rated strongly disagree to Strongly Agree) |

*Response options ranged from strongly disagree to strongly agree on 5 point ordinal rating scale.

** Reverse coded.
